# Prognostic Value and Immune Characterization of Genes Associated with Childhood Acute Leukemia applying Single-Cell RNA Sequencing

**DOI:** 10.2174/0118715303420113250818064855

**Published:** 2025-08-27

**Authors:** Zichao Lyu, Xiangyue Meng, Juan Xiao

**Affiliations:** 1 Department of Pediatrics, Peking Union Medical College Hospital, Chinese Academy of Medical Sciences and Peking Union Medical College, Beijing, 100730, China;; 2 Department of Breast Surgical Oncology, National Cancer Center/National Clinical Research Center for Cancer/Cancer Hospital, Chinese Academy of Medical Sciences and Peking Union Medical College, Beijing, 100730, China

**Keywords:** Childhood acute lymphoblastic leukemia, bulk RNA-seq, scRNA-seq, B cells, risk model, immune infiltration

## Abstract

**Introduction:**

Childhood acute lymphoblastic leukemia (cALL), the most common pediatric hematologic malignancy, arises primarily from B-cell origin and is strongly associated with immune dysfunction. This article integrated single-cell and bulk transcriptomic data to identify key B-cell subsets and cALL-related molecules as biomarkers.

**Methods:**

Single-cell RNA sequencing (scRNA-seq) Data from 2 pre-B high hyperdiploid (HHD) ALL patients and 3 healthy pediatric bone marrow samples (GSE132509) were utilized for cell clustering using the Seurat package. Functional enrichment, pseudo-time trajectory, and cell-cell communication analyses were performed using clusterProfiler, Monocle2, and CellChat R packages, respectively. Bulk RNA-seq data of 511 cALL samples in the TARGET-ALL-P2 cohort were used to construct a prognostic model *via* Cox and LASSO regression. Immune infiltration differences between different risk groups were analyzed using ESTIMATE, MCP-counter, and CIBERSORT algorithms.

**Results:**

The scRNA-seq analysis identified five cell subpopulations, with B cells demonstrating significant enrichment in cALL samples. Notably, the C2 subset was associated with cell proliferation. Ligand-receptor analysis revealed key interactions involving B cell C2. Four marker genes (*CENPF*, *IGLL1*, *ANP32E,* and *PSMA2*) were identified to build a risk model. Low-risk patients showed better survival, while high-risk patients had higher ESTIMATE scores.

**Discussion:**

This study examined the key role of B cells in cALL, constructed a risk model with strong prognostic predictive ability applying multi-omics analysis, and primarily explored its potential mechanism in immune regulation.

**Conclusion:**

This study revealed the critical role of B cells in cALL, and the prognostic model showed a high prediction accuracy, providing a potential target for individualized treatment of cALL.

## 1 INTRODUCTION

Childhood acute lymphoblastic leukemia (cALL), the most common hematological malignancy in children, arises from the clonal expansion of lymphoid progenitors that are arrested at specific differentiation stages [1, 2]. cALL accounts for approximately 75% of leukemia cases and nearly 30% of malignancies in children under 15 years of age [3]. cALL has B-cell and T-cell lineage origins, with B-cell origin predominating approximately 85% of cALL cases [4]. Constant improvement in diagnostic and treatment methods, such as multidrug combination therapy and hematopoietic stem cell transplantation, has significantly enhanced the survival rate of cALL, which now reaches 80-90% at 5 years [5, 6]. Nonetheless, chemotherapy resistance and recurrence still affect 15-20% of cALL patients, leading to a steadily rising death rate [7]. This underscores an imperative need to identify novel biomarkers and therapeutic targets that could improve the clinical outcomes for cALL.

B cell development begins with the differentiation of hematopoietic stem cells into common lymphoid progenitors, which then commit to the B cell lineage in the bone marrow [8]. In response to antigen exposure, common lymphoid progenitors primarily function as an early pool of B-cell progenitor cells that migrate to peripheral lymphoid tissues for functional activation after gradually differentiating through distinct intermediates [9]. Immunophenotypic analysis revealed that the fraction of B lymphocytes in fetal bone marrow cells is significantly larger than that in fetal liver and adult bone marrow, and it rapidly increases during mid-gestation [10]. This unique proliferative state of fetal bone marrow B progenitor cells is characterized by accelerated growth at the expense of differentiation within a specific developmental window, which may increase their susceptibility to malignant transformation [11]. Therefore, an in-depth understanding of the variability in B-cell suppression status and function in cALL patients is required to identify novel diagnostic and treatment targets for cALL.

Bulk RNA-seq or scRNA-seq have become powerful tools for analyzing gene expression profiles in tissue samples [12]. Bulk RNA-seq enables the discovery of biomarkers linked to different phenotypes of diseases [13] in ambient noise and resolves average gene expression in large tissue samples. On the other hand, scRNA-seq enables the characterization of gene expression at the single-cell level, facilitating cell-type-specific pathway analysis [14]. The immunological microenvironment of human malignancies has been extensively explored by numerous recent single-cell investigations [15]. This article aimed to investigate the key role of B cells in cALL and their impact on the prognosis. The study combined single-cell transcriptome and large-sample expression data to identify key B-cell subpopulations, screen signature genes, and construct a prognostic model for cALL. Overall, our study revealed the important role of B-cell-mediated immune mechanisms in cALL, providing a theoretical basis for precise prognostic assessment and individualized treatment strategies.

## 2 METHODOLOGY

### 2.1 Data Collection and Pre-processing

All analyses in this article were performed using publicly available datasets. First, we downloaded the bulk RNA-seq dataset of TARGET-ALL-P2 from the UCSC Xena database (https://xena.ucsc.edu/). The dataset was generated by the TARGET database (https://ocg.cancer.gov/programs/TARGET). Here, samples lacking clinical follow-up information or survival status were removed to ensure data integrity and analytical accuracy. Ensembl IDs in the gene expression matrix were then converted to their corresponding Gene Symbols. When multiple Gene Symbols corresponded to the same gene, the one with the highest expression value was retained for subsequent modeling and analysis. Finally, we divided the cALL 511 cancer samples into a training set (N = 358) and a test set (N = 153) at a ratio of 7:3 for subsequent studies.

### 2.2 Preprocessing of the scRNA-seq Data

The scRNA-seq dataset of cALL (GSE132509), which includes 2 cases of pre-B high hyperdiploid (HHD) ALL and 3 cases of healthy pediatric bone marrow mononuclear cell samples, was downloaded from the GEO database (https://www.ncbi.nlm.nih.gov/geo/). To pre-process the GSE132509 dataset, first, the samples were integrated by the anchoring method using the R package “Seurat” [16], and then the scRNA-seq data were filtered to retain high-quality cells with “nFeature_RNA > 300”, “nCount_RNA <= 100000”, and mitochondrial percentage below 15% (percent.mt < 15). Subsequently, the data were normalized using the NormalizeData function and filtered for highly variable genes using the FindVariableFeatures function. All genes were then normalized using the ScaleData function and linearly downscaled by the principal component analysis (PCA). To remove batch effects between samples, we used the Harmony package for integration analysis, specifying the variable group as the input. by.vars = “Samples”. The cell subpopulations were then grouped using the FindNeighbors and FindClusters functions at a resolution of 0.1 [17], following dimensionality reduction with the RunUMAP function [18]. The marker genes from the CellMarker 2.0 database were used to annotate each cell type [19].

### 2.3 Differential Gene Expression and Functional Annotation Analysis Between B-cell Subpopulations

Genes in B cells showing differential expression between cALL and control samples were screened by the “Seurat” R package’s FindMarkers function. B cells were further clustered using t-distributed Stochastic Neighbor Embedding (t-SNE) with the RunTSNE algorithm. Particularly highly expressed genes among various B cell subpopulations were selected using the FindAllMarkers function with a significance threshold of *p* < 0.05, to ensure the accuracy of cell annotation. Under the criteria of logfc.Threshold = 0.25, min.pct = 0.25; these highly expressed genes were employed as cellular markers. Kyoto Encyclopedia of Genes and Genomes (KEGG) enrichment analysis was performed on the upregulated genes using the R package “clusterProfiler” [20]. The enrichment analysis was conducted with a *p*-value threshold (*p*-value Cutoff) of 0.05 and a q-value threshold (qvalueCutoff) of 0.5, and the Benjamini-Hochberg method (pAdjustMethod = “BH”) was applied to control the false discovery rate and ensure statistical reliability.

### 2.4 Pseudo-time Trajectory Analysis

We performed pseudotime trajectory analysis using the R package “monocle2” [21]. Combined with the cell phenotypic data, we created the cds object using the newCellDataSet function, excluding genes expressed in fewer than ten cells. Differentially expressed genes (DEGs) between the cALL and control samples were selected using the differentialGeneTest tool. Next, dimensionality reduction (max_components =2, method = “DDRTree”) was performed, followed by pseudotime ordering *via* the orderCells function. Here, the trajectory root was assigned to the branch with the greater number of cells in the control samples.

### 2.5 Intercellular Communication

We analyzed intercellular communication using the “CellChat” R package and the CellChatDB.human database to examine ligand-receptor interactions between cell subpopulations in single-cell data [22].

### 2.6 Construction and Validation of Risk Models

Genes significantly associated with the prognosis of cALL in the TARGET-cALL training set (*p*<0.05) were identified from the specifically highly expressed DEGs in B cells between cALL samples and control samples by univariate Cox proportional risk regression analysis. Genes independently linked to the prognosis of patients in the TARGET-cALL training set were filtered by LASSO and stepwise regression analysis using the R package “glmnet” [23] and integrated into a Riskscore: Riskscore=Σβi×Expi. i refers to the gene expression, β is the Cox regression coefficient of the gene, and Expi indicates the expression of each gene. Each patient in both the validation cohort and the TARGET-cALL training cohort was assigned a Riskscore. Following normalization of the z-score, the TARGET-CALL patients were classified into low- and high-risk groups using the optimal threshold value of Riskscore. Survival analysis was conducted to compare the low- and high-risk groups using the R package “survminer” [24]. For prognostic analysis, a Kaplan-Meier (KM) survival curve was plotted, and the log-rank test was used to assess significant differences. Additionally, we evaluated the Riskscore performance using the R package “timeROC” [25] by calculating time-dependent area under the curve (AUC) values at 1-, 3-, and 5-year intervals in both the TARGET-cALL training and test sets.

### 2.7 Immune Infiltration Analysis of cALL

The R package “ESTIMATE” was applied to conduct immune infiltration analysis for samples in the TARGET-cALL training set to examine the correlation between Riskscore and immune function of cALL [26]. The “MCPcounter” package [27] was employed to determine the correlation between the Riskscore and the 10 immune cell scores of the TARGET-cALL training set. We obtained the gene signature matrix (LM22) for 22 immune cell subtypes from the official CIBERSORT website (https://cibersortx.stanford. edu/). The “CIBERSORT” package [28] in R was used to determine the infiltration of immune cells in the training set samples.

### 2.8 Statistical Analysis

R software (v4.1.2) was employed in all statistical analyses. Pearson’s correlation was used in correlation analysis. For comparisons between two groups, the chi-square test and the grouped t-test were used. Survival data were analyzed by applying Kaplan-Meier curves. Statistics were considered significant at *p*-value<0.05.

## 3 RESULTS

### 3.1 Identification of Cellular Subtypes in cALL

**Fig. (1):**
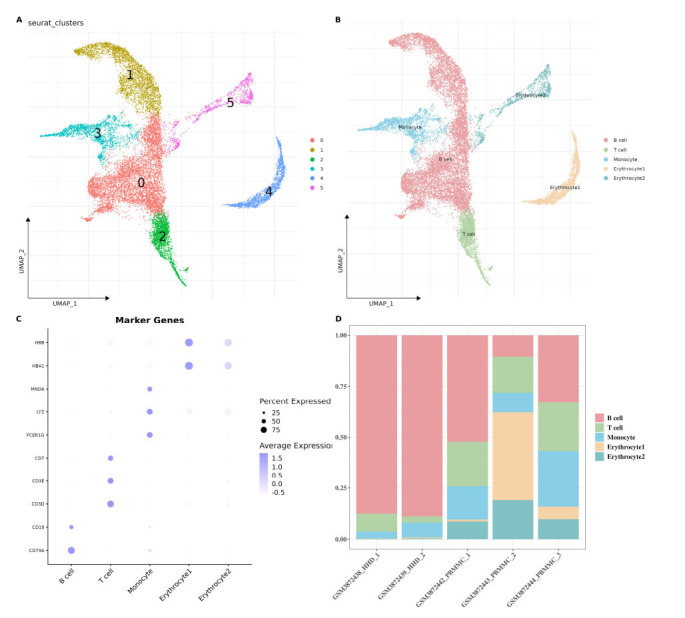
Based on scRNA-seq data in GSE132509 for cell subpopulation identification and annotation in cALL samples. (**A**) UMAP plot showing the distribution of six cell clusters obtained based on Seurat clustering analysis. (**B**) UMAP plot demonstrating cALL cell subpopulation annotation results, including B cells, T cells, monocytes, Erythrocyte1, and Erythrocyte2. (**C**) Expression bubble plots of representative marker genes for different cell subpopulations. (**D**) Histogram of the proportion of the composition of the various cell subpopulations in each sample.

Initial screening filtered a total of 15442 cells from the scRNA-seq data of the five samples in the GSE132509 dataset (Fig. **S1**). After normalization, downscaling and clustering analysis, six cell subpopulations were identified (Fig. **1A**). The classical maker genes were reclassified into five cellular subpopulations based on their expression in cell clusters, including B cell, T cell, Monocyte, Erythrocyte1 and Erythrocyte2 (Fig. **1B**). Subsequently, normalized expressions of representative marker genes of T cells (*CD3D*, *CD3E*, *CD7*), B cells (*CD19*, *CD79A*), Monocytes (*FCER1G*, *LYZ*, *MNDA*), Erythrocytes 1 (*HBA1*, *HBB*) and Erythrocytes 2 (*HBA1*, *HBB*) in the CellMarker2.0 database were visualized into bubble plot (Fig. **1C**). Finally, we compared the proportions of the 5 cell subpopulations in 2 cALL and 3 normal samples, respectively. As shown in the Figs. (**1D** and **B**) cells accounted for a significantly higher proportion in the cALL samples than in the control samples.

### 3.2 B Cell Heterogeneity in cALL

**Fig. (2):**
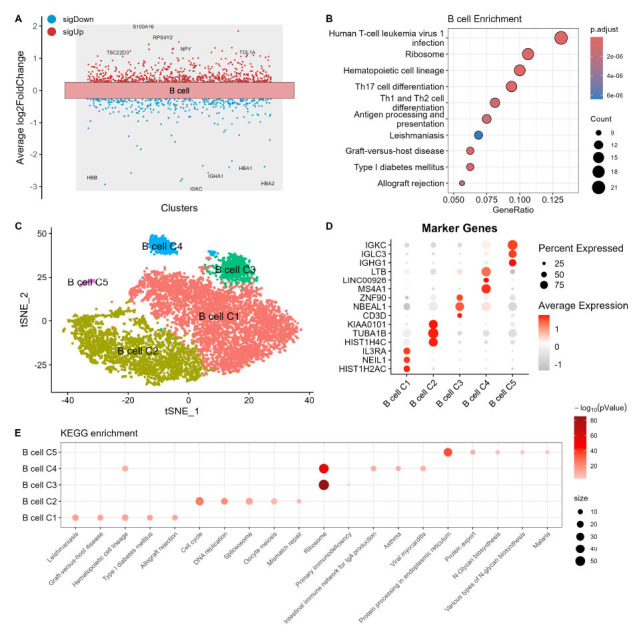
Identification of B-cell clusters based on scRNA-seq data from cALL patients. (**A**) The upper part shows the Log2 fold change distribution and top5 differential gene names of up-regulated differential genes in comparison between B-cell clusters, and the lower part shows the Log2 fold change distribution and top5 differential gene names of down-regulated differential genes in comparison between B-cell clusters. (**B**) Bubble plot of KEGG enrichment analysis of B-cell differential genes. (**C**) B-cell clustering annotated down-regulated t-SNE map. (**D**) B-cell different subpopulation-specific highly expressed genes. (**E**) KEGG functional enrichment of different subpopulation-specific highly expressed genes in B cells.

Next, the DEGs in B cells between cALL samples and control samples were analyzed. *S100A16*, *RPS4Y2*, *NPY*, *TSC22D3,* and *TCL1A* were the most significantly upregulated genes in B cells in the five samples. At the same time, HBA1, HBA2, IGHA1, IGKC and HBB were the most significantly downregulated genes in B cells (Fig. **2A**). To further explore the potential influence of B cells on cALL, enrichment analysis on the DEGs showed that these genes were enriched in pathways including ribosome and human T-cell leukemia virus 1 infection (Fig. **2B**). We further performed tSNE clustering on B cells. We categorized them into five B-cell subpopulations (Fig. **2C**). Fig. (**2D**) showed the expression of highly expressed genes in different subpopulations of B cells. KEGG analysis demonstrated that these genes were enriched in a variety of pathways, including the spliceosome, protein processing in the endoplasmic reticulum, and the cell cycle. In addition, B cell C2 was closely linked to cell cycle, DNA replication, the spliceosome, and other cellular value-added activities (Fig. **2E**).

### 3.3 Pseudo-time Trajectory Analysis

**Fig. (3):**
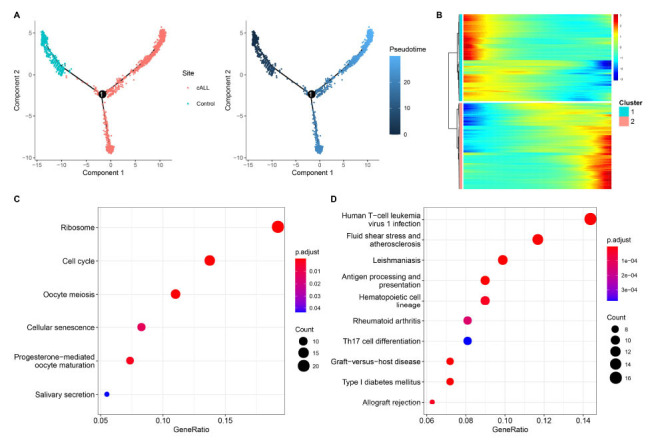
Pseudo-time trajectory analysis and enrichment analysis of B cell C2 in cALL. (**A**) Differentiation trajectory of B cell C2 cells evolving from normal to cALL samples. (**B**) Differential gene expression on the pseudo-time trajectory was categorized into 2 gene clusters based on differential expression trends. The color key, ranging from blue to red, indicates the relative expression level from low to high. (**C-D**) Cluster1 and Cluster2 KEGG enrichment analysis bubble plots.

To further explore the role of B cell C2 cells in the progression of cALL, we performed pseudo-time trajectory analysis (Monocle 2) to infer the pseudo-time developmental trajectory of B cell C2 cells. Here, we observed a progressive increase in the proportion of B cell C during the transformation from normal to cALL (Fig. **3A**). As shown in the Fig. (**3B**), the DEGs in B cell C2 in the proposed pseudo-time trajectories could be categorized into two clusters with distinct expression trends (Cluster 1 and Cluster 2). Further KEGG enrichment analysis showed that the genes in Cluster 1 were primarily enriched in ribosome and cell cycle pathways (Fig. **3C**). In contrast, the genes in Cluster 2 were mainly enriched in pathways related to antigen processing and presentation (Fig. **3D**).

### 3.4 Cellular Communication in cALL

**Fig. (4):**
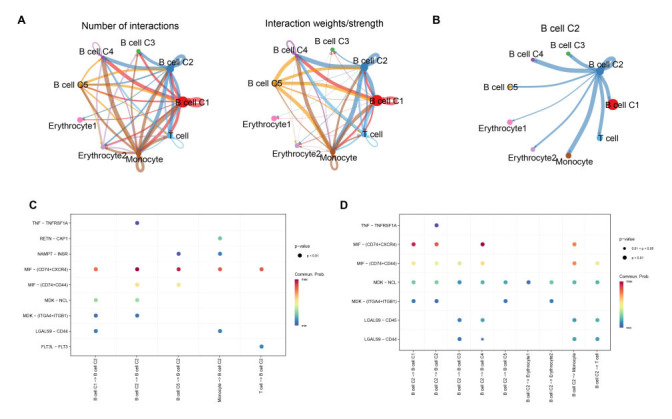
Communication analysis between different cell subpopulations in cALL. (**A**) Network diagram of cellular communication between cell clusters. The circle size is proportional to the number of cells in each cell taxon. The widths of the edges indicate the number of ligand-receptor pairs (left: shown by the “line” connecting the two cell types, the thicker the line, the higher the number of interaction pathways) and the strength of the communication (right: indicated by the “line” weight, the thicker the line, the stronger the interaction strength), respectively, between the different cell taxa. (**B**) Cell communication diagram of B cell C2 as a signal sender. (**C**) Bubble diagram of ligand-receptor acting on B cell C2 in other subpopulations. (**D**) Bubble diagram of B cell C2 subpopulation acting on ligand-receptors of different subpopulations.

By performing CellChat analysis, we constructed intercellular communication networks between most cells in cALL, including NK_T cells, Myeloid cells, Fibroblasts, and diverse subpopulations of tumor epithelial cells. It was observed that B cell C2 had the most intensive communication interactions among all cell subpopulations (Fig. **4A**). Subsequently, as shown in the Fig. (**4B**) cell C2 delivered significant signals to other cell subpopulations (*e.g.*, B cell C1, T cells, monocytes, *etc*.), further confirming its crucial role in the immune microenvironment of cALL. By extracting the ligand-receptor information of each cluster, we found that other cell subpopulations affected B cell C2 mainly through MIF - (CD74+CXCR4) receptor-ligand pair (Fig. **4C**), and that B cell C2 affected other cell clusters mainly through MDK-NCL (Fig. **4D**).

### 3.5 Establishment and Verification of the Prognostic Model

**Fig. (5):**
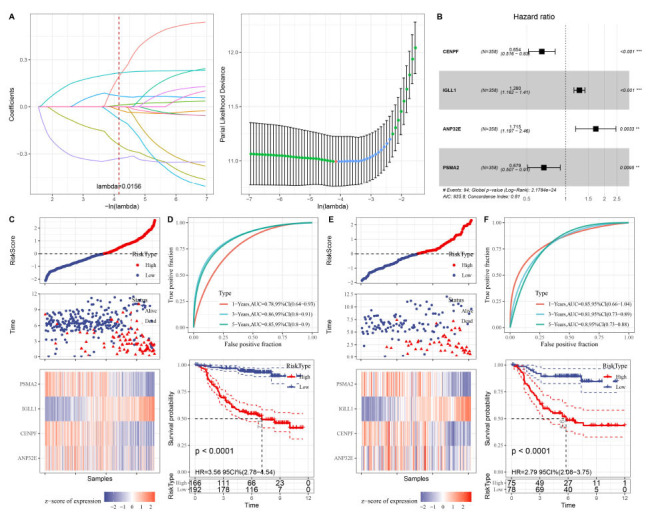
Validation of a four-gene prognostic model constructed based on B-cell-specific genes and its predictive effect in cALL. (**A**) Cross-validation results of LASSO regression screening for key prognostic genes. (**B**) Multivariate cox regression analysis of four characterized genes (*CENPF*, *IGLL1*, *ANP32E*, and *PSMA2*) and demonstration of risk ratios. (**C**) Risk score distribution, survival status, and four-gene expression heat maps in the training set. (**D**) Time-dependent ROC curves (1 year, 3 years, 5 years) and KM curves for four-gene models in the training set. (**E**) Score distribution, survival status, and four-gene expression heatmap of the risk model in the validation cohort. (**F**) Time-dependent ROC curves and survival analysis of risk models in the validation cohort.

There were 33 genes in the intersection between the marker genes of B cell C2 and the DEGs of B cells between cALL and control samples. These 33 genes in the TARGET-cALL training set were subjected to univariate Cox regression analysis, and 14 genes were identified that showed a strong impact on the prognosis of cALL (*p* < 0.05). After determining the optimal model at lambda=0.0156 with minimized error, we selected eight candidate genes (Fig. **5A**) and subsequently determine four prognostic genes (*CENPF*, *IGLL1*, *ANP32E* and *PSMA2*) in the TARGET-cALL training set by stepwise multifactorial regression analysis (Fig. **5B**). Based on the regression coefficients and the expressions of the four genes, Riskscore was formulated as: (-0.424*CENPF) + 0.247*IGLL1 + 0.54*ANP32E - 0.387*PSMA2. Patients in the TARGET-cALL training set were assigned to high-risk and low-risk groups based on the optimal critical value of Riskscore. Comparison of the survival differences between the two risk groups revealed a shorter survival in the high-risk group compared to the low-risk group (Fig. **5C**). In the TARGET-CALL training set, the high-risk patient group exhibited high expression of *IGLL1*. In contrast, the low-risk group had high expression of *CENPF*, *ANP32E* and *PSMA2* (Fig. **5C**). In the TARGET-cALL training cohort, the Riskscore reached 1-, 3- and 5-year AUC of prognostic classification of 0.78, 0.86 and 0.85, respectively (Fig. **5D**), indicating a strong performance of the model in the OS prediction for cALL patients. Furthermore, according to survival curve analysis, low-risk patients demonstrated a notably better OS than high-risk patients (*p* < 0.0001, Fig. **5D**). Consistently, the TARGET-cALL validation set cohort also showed similar outcomes, with patients in the cALL high-risk group having markedly worse survival (Fig. **5E**). *CENPF*, *ANP32E* and *PSMA2* exhibited minimal expression in the high-risk group, whereas *IGLL1* was high-expressed (Fig. **5E**). The OS of patients in the cALL high-risk group was significantly worse than the low-risk group (*p* < 0.0001, Fig. **5F**). In the TARGET-cALL validation set, 1-, 3- and -5-year AUC value reached 0.85, 0.81 and 0.8, respectively (Fig. **5F**). In addition, we further validated the performance of the Riskscore in the TARGET-ALL-P2 full cohort (n=511) (Fig. **S2A**). The results showed that the model had an AUC of 0.81, 0.84, and 0.84 for 1-, 3-, and 5-year survival prediction, respectively (Fig. **S2B**). The OS of high-risk patients was significantly lower than that of the low-risk group (Fig. **S2C**). These results indicated that the model had strong prognostic predictive ability and stability in the integrated cohort.

### 3.6 Risk Modeling and Immune Microenvironment

**Fig. (6):**
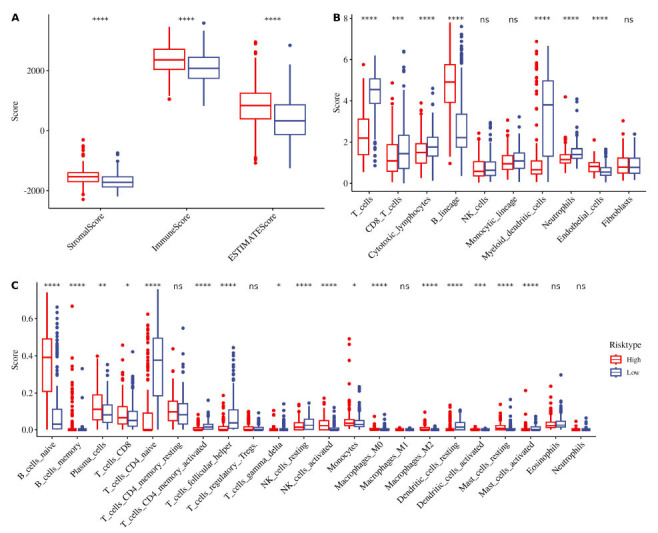
Relationship between risk score and immune cell infiltration in cALL patients. (**A**) Immune infiltration was calculated by the ESTIMATE method between different risk groups. (**B**) MCP-counter to assess the difference in immune cell scores between high and low risk groups. (**C**) CIBERSORT assesses the difference in immune cell infiltration between high- and low-risk groups. Ns represents *p* > 0.05; *p < 0.05, **p < 0.01, ***p < 0.001, and ****p < 0.0001.

The difference in the immune milieu between patients in the low- and high-risk subgroups of the TARGET-cALL training cohort was examined to clarify the relationship between the Riskcore and immune microenvironment in the two risk groups of cALL. We observed higher immune cell infiltration in high-risk cALL patients who demonstrated notably higher ESTIMATEScore ImmuneScore—Stromalscore than the low-risk group (Fig. **6A**). The MCP-counter evaluation of immune cell infiltration revealed that the low-risk group exhibited a higher abundance of myeloid dendritic cells, T cells, CD8 T cells, cytotoxic lymphocytes, and neutrophils compared to the high-risk group. In contrast, the low-risk group exhibited a lower infiltration abundance of B_lineage and Endothelial cells (Fig. **6B**). Analysis of 22 immune cell types revealed that the high-risk group had higher levels of B_cells_naïve, Mast_cells_resting, and Macrophages_M2 immune cell infiltration than the cALL low-risk group. The low-risk group of cALL displayed increased infiltration of immune cells, including T cells (follicular helper, CD4 naïve), and resting dendritic cells (Fig. **6C**).

## 4 DISCUSSIONS

Research has shown that cALL patients exhibit impaired B-cell immune responses, including reduced B-T cell interaction. This is primarily caused by the malignant expansion of B-cells that suppresses normal B-cell function and inhibitory cytokines, leading to B-cell hyperactivity [29-31]. The expression level of fetal B progenitor cells is higher than that of postnatal progenitor cells. Additionally, chromosomal translocations linked to cALL are influenced by genes specific to B cells [32]. CALL is characterized by multiple genetic lesions that affect both the cell cycle and B cell development [31]. These findings all indicated crucial functions of B cells in the emergence of cALL. The current study conducted a comprehensive analysis of cALL samples and discovered that B-cell clusters were a major cellular component in these samples. Importantly, we found that the B cell C2 subpopulation was closely linked to proliferative activity in cALL and played a crucial role in immune regulation through specific ligand-receptor pairs. We developed a robust prognostic signature with four B-cell-specific genes (*CENPF*, *IGLL1*, *ANP32E,* and *PSMA2*C), which demonstrated consistent predictive accuracy across both training and validation cohorts. These B-cell-specific genes can be considered as promising biomarkers for the risk stratification and potential therapeutic targets in cALL treatment.

The KEGG enrichment analysis revealed that the DEGs in B cells between cALL and control samples were mainly enriched in pathways including ribosome and human T-cell leukemia virus 1 (HTLV-1) infection, consistent with previously reported high HTLV-1/2 prevalence in ALL patients [33, 34]. Notably, HTLV-1 encodes proteins that transcriptionally and post-transcriptionally inhibit B-cell leukemia/lymphoma 11B tumor suppressor genes [35]. Researchers have discovered that aberrant expression of ribosomal proteins may contribute to the heterogeneity of cALL by hindering the normal development of leukemia cells [36]. Additionally, our investigation verified that some highly expressed genes in certain B-cell subpopulations were strongly linked to cellular value-added functions, including spliceosomes and DNA replication [37].

ROC curves demonstrated that the four genes were associated with the prognosis of cALL patients and could accurately predict their prognosis. Tumors often develop drug resistance, which shortens patients’ survival and causes early relapse. A poor prognosis for patients who received chemotherapy is found to be associated with highly expressed *CENPF*, an important regulator of cell cycle progression in triple-negative breast cancer [38]. A study reported that the IGLL1 variant causes agammaglobulinemia, which has been traditionally considered a rare and severe B-cell deficiency [39]. In T-ALL, under the regulation of *FYB1, IGLL1* is a super-enhancer driver gene that plays a crucial role in maintaining tumor cell self-renewal and inhibiting apoptosis [40]. This suggested that *IGLL1* may have different biological functions in different immune profiles and diseases. Notably, this study found that *IGLL1* was highly expressed in cALL, predominantly in the B-cell lineage, and that its high expression was closely associated with a poor prognosis. This suggested that *IGLL1* may play a potential role in promoting cell proliferation or maintaining an immature state in B-cell leukemia in cALL. Therefore, the function of *IGLL1* may be cell lineage-dependent, but its specific mechanism requires further investigation using a larger sample size and experimental validation. By preventing ferroptosis in esophageal cancer cells, *ANP32E*, a member of the acidic nuclear phosphoprotein 32 family, stimulates tumor cell growth and enhances treatment resistance in cancer cells [41]. Lower expression of *ANP32B*, another member of this family, is linked to a poor prognosis of patients with B-cell ALL [42]. Karyotypic abnormalities in cancer are considered acquired primary chromosomal abnormalities. Karyotypic abnormalities also play a critical role in the progression of certain malignancies, often resulting in the production of chimeric genes that are significant for prognosis, diagnosis, and pathogenicity. *PAN3*-*PSMA2* fusion transcripts crucially contribute to the development of acute myeloid leukemia in myelodysplastic disorders [43]. These findings indicated that the four genes identified in our study may be involved in the key biological processes related to cALL progression and prognosis, supporting their potential relevance as biomarkers in cALL.

The immune system plays a complex and dual role in tumor development. On the one hand, various immune cells can promote inflammation and carcinogenesis by participating in inflammatory processes [44]. On the other hand, immune surveillance helps eliminate abnormal cells [45]. Tumors utilize complex signaling networks to establish immunosuppressive microenvironments, which enable immune evasion [46]. In the context of cALL, immune dysfunction is associated with increased mortality and relapse [47]. In our study, high-risk cALL patients exhibited higher StromalScore, ImmuneScore, and ESTIMATEScore, suggesting the presence of a distinct immune microenvironment. However, whether such increased immune cell infiltration reflected functional immune activation or immunosuppression remained unclear. Prior studies have reported that leukemia cells may secrete inhibitory cytokines or interfere with the differentiation and proliferation of immune cells [48]. In murine models, robust T cell activation is associated with sustained leukemia remission, whereas T cell depletion following chemotherapy may increase relapse rates [49]. In clinical settings, cALL patients often develop severe neutropenia during induction therapy, and neutrophil-related markers have been proposed as indicators for infection risk [50]. Moreover, B-cell hyperplasia can facilitate cALL progression in specific anatomical sites [51]. Together, these results indicated that the immune microenvironment varied between different cALL risk groups, but further mechanistic studies are needed to clarify its functional implications.

## CONCLUSION

Based on scRNA-seq and bulk RNA-seq data, this study focused on pediatric Pre-B HHD-ALL and identified five major cell subpopulations, with significant enrichment of leukemic B cells. The C2 subset was associated with cell proliferation and interacted with immune cells *via* specific ligand-receptor pairs. A prognostic model was constructed using four key B-cell-specific genes (CENPF, IGLL1, ANP32E, and PSMA2) and demonstrated high accuracy in risk stratification and survival prediction in cALL, providing insights into cALL heterogeneity and prognosis.

## LIMITATIONS

This study had several limitations that should be acknowledged. The small scRNA-seq sample size (two cALL patients and three healthy controls) may not fully represent the cellular heterogeneity and immune characteristics of cALL, necessitating larger multi-center studies for further verification. Key clinical variables such as gene mutation status and treatment regimen were not included, which potentially limited the control of potential confounding factors. Our future study will incorporate multivariate analysis to improve the clinical applicability of the model. In addition, the molecular subtype heterogeneity within cALL was not fully explored, which requires stratified analyses based on subtype information to uncover the specific mechanisms. Notably, while the computational findings were promising, experimental validation through qRT-PCR and functional assays is warranted. Moreover, external validation relied solely on the TARGET-ALL-P2 dataset due to the limited availability of publicly accessible cALL cohorts with complete clinical follow-up, underscoring the importance of additional independent validation to assess the model's generalizability. Finally, the technical limitations of scRNA-seq, including expression bias and noise affecting the detection of low-abundance genes, should be addressed by integrating noise reduction algorithms with emerging spatial transcriptomic technologies for improved resolution and cellular localization.

## Data Availability

The datasets generated and/or analyzed during the current study are available in the (GSE132509) repository, (https://www.ncbi.nlm.nih.gov/geo/query/acc.cgi?accGSE132 509).

## LIST OF ABBREVIATIONS

cALL: Childhood Acute Lymphoblastic Leukemia
scRNA-seq: Single-cell RNA Sequencing
Bulk RNA-seq: Bulk RNA Sequencing
LASSO: Least Absolute Shrinkage and Selection Operator
TARGET: Therapeutically Applicable Research to Generate Effective Treatments
OS: Overall Survival
PCA: Principal Component Analysis
GEO: Gene Expression Omnibus
KEGG: Kyoto Encyclopedia of Genes and Genomes
ROC: Receiver Operating Characteristic Analysis
AUC: Area Under the ROC Curve
