# Supplementary material for: Prognostic Value and Immune Characterization of Genes Associated with Childhood Acute Leukemia applying Single-Cell RNA Sequencing
Source: Endocr Metab Immune Disord Drug Targets. 2025 Aug 27;26:E18715303420113. doi: 10.2174/0118715303420113250818064855 (PMC13334267; doi:10.2174/0118715303420113250818064855)
Supplement: Supplementary file 1 [file EMIDDT-26-E18715303420113_SD1.pdf]

Supplementary Material

Prognostic Value and Immune Characterization of Genes Associated with Childhood Acute Leukemia applying Single-Cell RNA Sequencing

Zichao Lyu[1,*], Xiangyue Meng[2] and Juan Xiao[1,*]

[1]Department of Pediatrics, Peking Union Medical College Hospital, Chinese Academy of Medical Sciences and Peking Union Medical College, Beijing, 100730, China; [2]Department of Breast Surgical Oncology, National Cancer Center/-National Clinical Research Center for Cancer/Cancer Hospital, Chinese Academy of Medical Sciences and Peking Union Medical College, Beijing, 100730, China

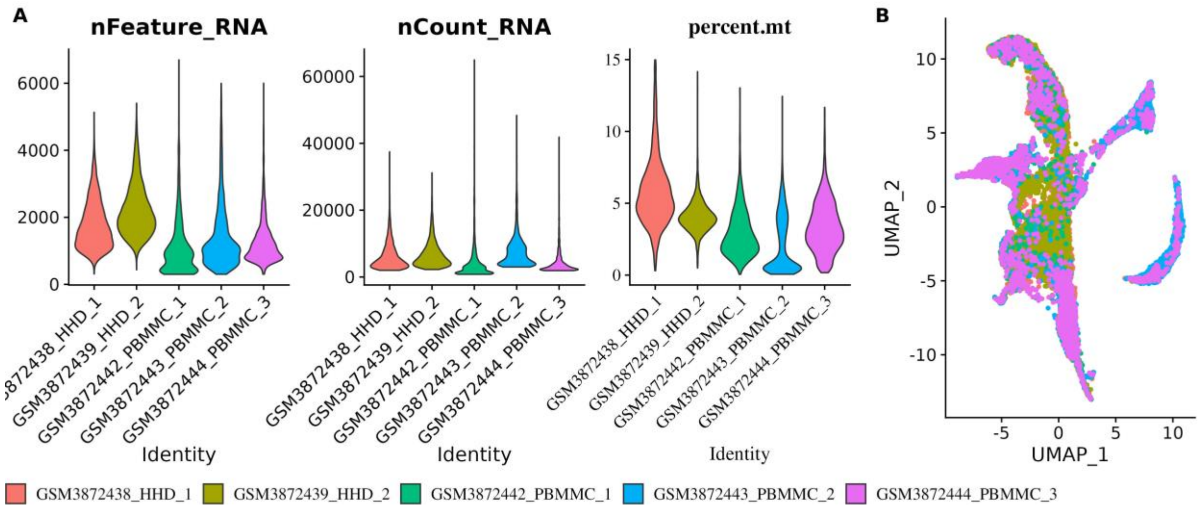

Supplementary Fig. (S1). scRNA-seq data reprocessing results for the GSE132509 dataset. (A) Relationship between mRNA, UMI, mitochondrial content and rRNA content for each sample after filtering. (B) Sample distribution plots for principal component analysis downscaling.

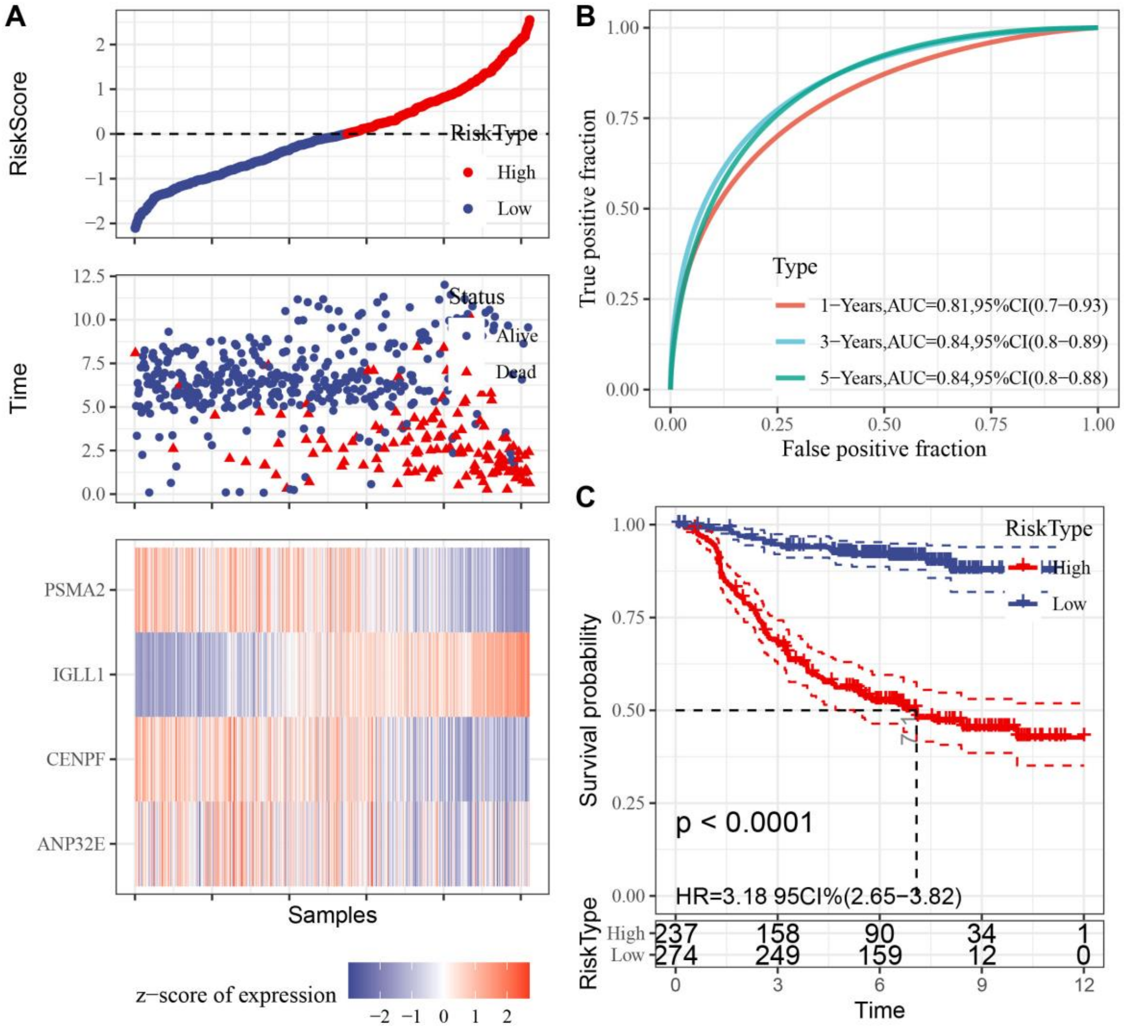

Supplementary Fig. (S2). Based on the TARGET-ALL-P2 full cohort (n=511) in order to validate the risk model. (A) Risk score distribution, survival status, and four-gene expression heatmap for Risk modeling. (B) Time-dependent ROC curves (1, 3, and 5 years) for the four-gene (CENPF, IGLL1, ANP32E and PSMA2) risk model. (C) Kaplan-Meier survival curve analysis of patients in high and low risk groups.
